# Codifying Physicians’ Fiduciary Duty to Rebuild Patient-Physician Trust in China

**DOI:** 10.34172/ijhpm.9879

**Published:** 2026-07-22

**Authors:** Lei Zhao, Yue Wang

**Affiliations:** School of Law, Ocean University of China, Qingdao, China.

## Introduction

 Trust is the foundation on which the effective delivery of medical care rests. Illness places patients in a position of vulnerability, and the uneven distribution of medical knowledge produces a marked information asymmetry between physician and patient. Because patients must rely on physicians throughout diagnosis and treatment, the relationship is bound together by trust.^1^ Patients need to believe that the physician acts in their interest, and this belief leads them to describe their condition truthfully, to follow the treatment plan, and to cooperate with care—all preconditions for diagnosis and treatment to proceed.^2^ Where trust is absent, patients conceal their condition, refuse advice, and adopt a defensive posture, harming the interests of both parties. Low trust also gives rise to frequent medical disputes and can aggravate violence directed at medical personnel.^3^

 In receiving care, patients must trust the physician both to manage their health and to safeguard their financial interests by avoiding unnecessary expenditure. Medical care is a credence good: before and after treatment, patients are generally unable to judge whether a service is necessary or whether its quality meets the appropriate standard, and can only rely on the professional who holds both the diagnostic and the therapeutic role.^4^ Because the physician both recommends treatment and stands to gain from it, a risk of overtreatment and unreasonable charges follows. For this reason the physician has long been regarded as owing a duty of loyalty to the patient.^5^ A fiduciary duty arises when one party, by virtue of knowledge, power, or professional skill, provides a service to a more vulnerable party and thereby assumes an obligation to act with loyalty and good faith, placing the other party’s interests above personal gain or institutional pressure. The patient-physician relationship has long satisfied this definition.^6^

 The structural transformation of China’s medical system has placed this relationship of trust under sustained strain. That transformation, though profound, has produced an unforeseen problem: a serious crisis of trust between physicians and patients. A study of several large hospitals in China reports that patient trust in medical personnel is on the whole low to moderate, and a number of surveys show that severe distrust has already provoked extensive workplace violence against physicians.^7,8^ China is also among the few countries that require extensive and stringent security-screening facilities in hospitals.^9^

 The roots of patient-physician conflict and distrust in China can be traced to the market-oriented health reforms launched in the 1980s. As government fiscal subsidies contracted, medical institutions were forced to rely on their own revenue to sustain operations; the practice of funding medical services through drug sales took hold; and the provision of care was gradually distorted into a form of commercial transaction.^10^ The current round of reform, exemplified by the Sanming model now being promoted nationwide, seeks to reverse this commercial orientation.^11^ Yet the distortions in professional culture produced by decades of marketization are difficult to repair fully through structural and institutional adjustment alone.

 This article reviews the commercialization of China’s medical sector, the crisis of patient-physician trust and violence against physicians, fiduciary-law theory, and comparative medical law, drawing on China’s current laws and regulations together with representative case law and professional norms from Anglo-American and continental European jurisdictions. The sources were selected purposively for their conceptual relevance rather than through a systematic search, and the evidence base is accordingly illustrative rather than exhaustive. The analysis is primarily normative and policy-oriented. We argue that legislation establishing fiduciary legal liability can provide important legal support for the Sanming reform: by defining the physician’s fiduciary duty in statute, it offers a clear legal signal, builds an ex ante regulatory framework, and helps restore trust.

## From Healing to Commerce

 Before the 1980s, China’s medical system operated on a public-welfare model that, despite scarce resources, sustained trust through visible institutional commitment to community health; the “barefoot doctors” exemplified medicine as public service.^12^ Marketization reversed these incentives. Reduced subsidies forced institutions to generate revenue, creating structural pressures to overprescribe, over test, and favor higher-margin interventions regardless of medical necessity.^13^ Physicians were repositioned from patient advocates toward institutional revenue generators; some accounts describe the former “barefoot doctors” as having become a higher-income group whose earnings may include gray income such as informal “red packet” payments.^14^

 These changes had lasting effects. Patients increasingly read clinical recommendations as commercial maneuvers, breeding suspicion that has manifested in confrontation and violence.^15^ Empirical work links perceived economic exploitation to patient dissatisfaction, and a self-reinforcing cycle has emerged: suspicion prompts defensive medicine, which inflates costs and deepens the belief that patients are being economically exploited.^16^

## Inadequacies in the Current Legal Framework

 The Physicians Law (2021) requires physicians to observe professional ethics, serve patients conscientiously, and refrain from exploiting their position for illegitimate gain; Article 31 sets out the physician’s core practice obligations.^17^ Yet these duties operate primarily as administrative and disciplinary obligations rather than as enforceable, patient-facing duties of loyalty carrying civil remedies.

 The medical-malpractice tort system requires plaintiffs to prove breach of a medical standard and a causal link to measurable physical harm. It is therefore ill-suited to conflicts of interest that cause economic harm without quantifiable physical injury—for example, arranging unnecessary but profitable procedures, or prescribing higher-margin drugs where equally effective low-cost alternatives exist. Such conduct injures patients’ economic interests and corrodes trust, yet often falls outside tort remedies, leaving a regulatory gap. The Regulations on the Prevention and Handling of Medical Disputes provide procedures that are reactive and cumbersome, addressing disputes after they arise rather than establishing proactive standards.^18^

## Fiduciary Duty: A Mechanism for Restoring Trust

 As noted, healthcare services are credence goods: patients cannot readily assess the necessity or quality of treatment before or after it is rendered, which compounds vulnerability and limits the disciplining force of voluntary self-regulation.^4,19^ Because the service is trust-dependent in its very structure, many countries have developed fiduciary duties to address such asymmetries by imposing enforceable obligations of loyalty, disclosure, and care.^6^

 Fiduciary liability is a specialized legal regime designed to address problems of trust, and it has proven effective in corporate governance and other fiduciary contexts.^20^ In healthcare, the law explicitly recognizes that physicians bear fiduciary obligations. U.S. law, for instance, recognizes the physician’s fiduciary role: *Canterbury v. Spence* established patient-centered disclosure standards,^21^ and *Moore v. Regents of the University of California* required disclosure of physician conflicts of interest, including economic ones.^22^ The American Medical Association’s Code likewise places patient welfare above self-interest.^23^ Comparable protections exist elsewhere: the U.K. Supreme Court’s *Montgomery v. Lanarkshire Health Board* adopted a patient-centered disclosure standard,^24^ while Germany and France achieve similar ends through good-faith contract principles and statutory patients’-rights frameworks.^25^ Across these jurisdictions, the law recognizes that asymmetry and vulnerability warrant enforceable, patient-protective duties. The effectiveness of fiduciary obligations nonetheless varies across countries, owing chiefly to differing national conditions and degrees of enforcement.

## Codifying Fiduciary Duty Under the Sanming Reform

 The Sanming reform offers an opportunity to rebuild a trust-based patient-physician relationship. We propose that physicians’ fiduciary duty be codified as an amendment to Article 31 of the Physicians Law of the People’s Republic of China, supplementing that provision’s administrative obligations with an explicit, civilly enforceable duty of loyalty and disclosure owed to patients. Locating the reform in a specific provision identifies where it should enter the existing framework; illustrative draft text is set out in Supplementary file 1.

###  A. Expressive Function and Systematic Reform Signaling: Correcting Over-Commercialized Healthcare Service Models

 The erosion of trust within China’s healthcare system resulted from the transition toward a profit orientation since the 1980s. Numerous medical professionals received training and practice within this commercialized, profit-driven system, with long-term exposure to such environments significantly affecting their professional spirit and practice approaches. Improving patient-physician relationships and increasing trust requires establishing clear, legally binding fiduciary legal systems to correct these misaligned priorities. Legislation establishing fiduciary duty would have a powerful expressive effect, marking a fundamental transformation in healthcare policy.^26^ Formally recognizing fiduciary duty would communicate a clear signal that healthcare is returning to the principle of placing patient health interests first, restoring the fundamental public service attributes of medical practice.

###  B. Rebalancing Power and Patient Agency

 Voluntary self-regulation has failed under strong economic incentives. By granting patients enforceable claims when loyalty duties are breached, a fiduciary standard would empower the more vulnerable party and foster more collaborative care.^27^ Trust is more likely to emerge when relationships are balanced and subject to mutual checks. The standard would not curtail legitimate clinical autonomy but would mark its boundaries where economic incentives threaten clinical judgment.

###  C. Protecting Clinical Judgment While Penalizing Self-dealing

 A central objection to legal duties in medicine is that they may chill the clinical autonomy on which good care depends. Corporate fiduciary law has long resolved an analogous tension and offers a workable template. Under the business judgment rule, courts presume that a manager acted in good faith and on an informed basis and decline to second-guess honest professional judgments; liability attaches only when a plaintiff rebuts that presumption by showing self-dealing, bad faith, or fraud, at which point the burden shifts to the fiduciary to prove the transaction was entirely fair.^28^ A codified physician fiduciary duty should mirror this structure. Bona fide clinical decisions, including reasonable diagnostic and treatment choices that later prove mistaken, would retain full protection; a physician would not be liable merely because an intervention failed or an alternative existed. The duty would bite only on defined categories of self-interested conduct that no clinical rationale can justify: accepting pharmaceutical or device kickbacks, self-referral to facilities in which the physician holds an undisclosed financial interest, soliciting informal payments (“red packets”) from patients, and overtreatment or overdiagnosis undertaken for revenue rather than medical need.

 Calibration of the trigger is important. To preserve clinical freedom and avoid litigation over ordinary judgment, civil liability could attach only where conduct crosses clear thresholds—for example, solicited payments above a defined sum, or economically unnecessary care imposing costs above a defined sum on the patient—with the specific figures set by the legislature or implementing regulation; below these thresholds, professional and administrative discipline would remain the primary channel. Once a threshold breach is shown, the corporate model supplies the remedies: disgorgement of the improperly obtained gain, which reaches kickback and red-packet income even absent provable physical harm; compensatory damages for the patient’s economic loss; and, for egregious or repeated misconduct, enhanced damages and referral for licensing discipline.^29^ Disgorgement is well suited to this setting because it returns the fruits of disloyalty regardless of whether physical injury occurred, closing the remedial gap that tort law leaves open.

###  D. Filling the Remedial Gap

 A fiduciary standard would attach liability to disloyalty itself, independent of proof of physical harm, providing remedies for economic exploitation that tort law misses. Because dissatisfaction correlates more strongly with perceived economic exploitation than with perceived clinical incompetence,^16^ aligning remedies with this source of grievance would strengthen both legal protection and system legitimacy.

###  E. Trust Reconstruction

 Clear standards give physicians principled grounds to resist inappropriate institutional pressure and give patients legal channels in place of confrontation, converting potential interpersonal conflict into structured dispute resolution. Over time, accessible remedies can support a virtuous cycle in which legal protection enhances confidence and reduces friction.

## Conclusion

 Codifying physicians’ fiduciary duty in statute would reorient healthcare governance toward medicine’s public-service mission. The persistent erosion of the patient-physician relationship reflects institutional failures that voluntary codes and incremental tort adjustments cannot resolve. Enacted through an amendment to Article 31 of the Physicians Law, an explicit and enforceable fiduciary duty would provide both an immediate corrective and a longer-term commitment to re-centering patient welfare. The aim is not to restore an earlier model but to build new institutional arrangements that reconcile efficiency with the ethics of care. Codification is one valuable component of trust rebuilding rather than a definitive solution; embedding a fiduciary duty in the legal framework is a necessary step toward the trust on which equitable, effective, and humane healthcare depends.

## Disclosure of artificial intelligence (AI) use

 Not applicable.

## Ethical issues

 We confirm that the research, analysis, arguments, and conclusions presented in this viewpoint are the authors’ own. AI tools were used solely to assist with language editing; they were not used to generate the study’s ideas, claims, or scholarly content.

## Conflicts of interest

 Authors declare that they have no conflicts of interest.

## Supplementary files


Supplementary file 1. Illustrative Draft Amendment to Article 31 of the Physicians Law.
