# Supplementary material for: Codifying Physicians’ Fiduciary Duty to Rebuild Patient-Physician Trust in China
Source: Int J Health Policy Manag. 2026 Jul 22;15:9879. doi: 10.34172/ijhpm.9879 (PMC13770237; doi:10.34172/ijhpm.9879)
Supplement: Supplementary file 1 [file ijhpm-15-9879-s001.pdf]

Article title: Codifying Physicians' Fiduciary Duty to Rebuild Patient-Physician Trust in China

Journal name: International Journal of Health Policy and Management (IJHPM)

Authors' information: Lei Zhao*, Yue Wang*

School of Law, Ocean University of China, Qingdao, China.

***Correspondence to:** Lei Zhao; Email: zl@ouc.edu.cn & Yue Wang, Email: wangyues@bjmu.edu.cn

Citation: Zhao L, Wang Y. Codifying physicians' fiduciary duty to rebuild patient-physician trust in China. Int J Health Policy Manag. 2026;15:9879. doi:10.34172/ijhpm.9879

Supplementary file 1. Illustrative Draft Amendment to Article 31 of the Physicians Law

(1) Physicians shall not take advantage of their positions to demand or illegally accept money or property, or seek other illegitimate benefits; nor shall they subject patients to unnecessary examinations or treatments.

(2) In the practice of medicine, a physician owes the patient a duty of loyalty and good faith. The physician shall act in the patient's best interests and shall not allow personal financial interest, or the financial interest of any institution or third party, to override the patient's interest in appropriate care.

(3) A physician shall disclose to the patient any personal or institutional financial interest that could reasonably be expected to influence a clinical recommendation, including any interest in a facility, product, or service to which the patient is referred.

(4) A clinical decision made in good faith and on a reasonable professional basis does not breach the duty under this Article, even if the decision later proves mistaken or an alternative course existed. A physician shall not be civilly liable under this Article for an exercise of professional judgment that meets this standard.

(5) A physician breaches the duty under this Article by: (a) demanding or accepting a kickback, commission, or other improper benefit in connection with the prescription, supply, or referral of a drug, device, examination, or treatment; (b) referring a patient to a facility, product, or service in which the physician holds an undisclosed financial interest; (c) soliciting or accepting an informal payment from a patient or a patient's family the value of which exceeds the threshold set by the State Council's health administration department; or (d) Ordering or performing examinations or treatments without medical indication for the purpose of financial gain, where the resulting unnecessary costs to the patient significantly exceed the DIP (Diagnosis-Intervention Packet) standard. A patient harmed by such a breach may bring a civil action. The court may order disgorgement of the improper benefit obtained by the physician, compensation for the patient's economic loss, and, in cases of serious or repeated breach, additional liability as provided by law. Liability under this Article is independent of, and additional to, any liability for medical malpractice and any administrative or disciplinary sanction.
